# A new species of *Pheidole* (Formicidae, Myrmicinae) from Dominican amber with a review of the fossil records for the genus

**DOI:** 10.3897/zookeys.866.35756

**Published:** 2019-07-24

**Authors:** Alexandre Casadei-Ferreira, Julio C.M. Chaul, Rodrigo M. Feitosa

**Affiliations:** 1 Laboratório de Sistemática e Biologia de Formigas, Departamento de Zoologia, Universidade Federal do Paraná, Avenida Francisco Heráclito dos Santos, s/n, Centro Politécnico, Mailbox 19020, 81531-980, Curitiba, Brazil Universidade Federal do Paraná Curitiba Brazil; 2 Pós-Graduação em Ecologia, Departamento de Biologia Geral, Universidade Federal de Viçosa, 36570-900, Viçosa, MG, Brazil Universidade Federal de Viçosa Viçosa Brazil

**Keywords:** Miocene, morphological diversity, new status, taxonomy

## Abstract

*Pheidole* comprises approximately 1,000 extant species distributed worldwide, being particularly diverse in the New World. In addition to its high diversity and ecological prevalence, the genus is also characterized by the predominantly intraspecific dimorphism, with major and minor workers. Currently, five fossil species are known, all of which are represented only by minor workers. A new species, †*Pheidoleanticua***sp. nov.**, is described from Dominican amber, based on a major worker. Additionally, the identity of the currently known fossil species in *Pheidole* is discussed and †*P.cordata* from Baltic amber is considered as incertae sedis, resulting in no *Pheidole* species currently recognized for Baltic amber

## Introduction

*Pheidole* Westwood 1839 is the largest myrmicine ant genus with 1,047 species worldwide (Bolton 2019). Species in this genus are generally characterized by conspicuous dimorphism, with major and minor workers. Currently, five fossil species of *Pheidole* are known: †*Pheidoleprimigenia* Baroni Urbani, 1995 and †*Pheidoletethepa* Wilson, 1985 from Dominican amber (Early Miocene) dated from 16–19 mya (Seyfullah et al. 2018); †*Pheidoletertiaria* Carpenter, 1930 based on compression fossils from the Florissant in Colorado (Late Eocene) dating from 34.07 ± 10 mya (Evanoff et al. 2001); †*Pheidolerasnitsyni* Dubovikoff, 2011 originally described as a Baltic amber fossil, but now recognized as a copal inclusion (Perkovsky 2016); and †*Pheidolecordata* (Holl 1829) described from the Baltic amber (Late Eocene) dating from 34–48 mya (Seyfullah et al. 2018). All fossil records mentioned for *Pheidole* so far are exclusively represented by minor workers. Here we describe a new species of *Pheidole* for the Dominican amber based on a major worker. We also propose changes to the status of the other fossil species in the genus.

## Material and methods

The studied inclusion was originally immersed in a 26 × 14 × 14 mm, orange, oval Dominican amber piece with a fragmentary specimen of Psocoptera as a syninclusion, which was lost after treatment of the stone. This piece was faceted and polished for better visualization using increasingly finer sandpapers and, lastly, liquid silver polishing on a soft, clean, and dry cloth. The specimen was bought from the eBay store “ambergalleryboutique1” in July 2017. The seller confirmed that the specimen was mined in “La Toca” site. The specimen had the morphospecies code “*Pheidole* ufv-65” from 2017 to 2019 on Antweb.

The holotype is deposited at the Padre Jesus Santiago Moure Entomological Collection of the Universidade Federal do Paraná, Curitiba, Brazil (**DZUP**). Observations were made at 80× magnification with a Zeiss SteREO Discovery.V8 dissecting microscope. Measurements were made with a dual-axis micrometer stage with output in increments of 0.001 mm. All measurements are given in mm. The high-resolution images were made with an Axiocam 305 color camera coupled to a Zeiss SteREO Discovery.V20. Extended depth focus was made with Zen Blue v.2.3 and subsequently treated to correct for brightness and contrast. Digital vectorization was based on original photographs.

We adopted morphological terminology and measurements proposed by Longino (2009) and sculpture terminology by Harris (1979).

## Results

### † Pheidole anticua sp. nov.

Taxon classificationAnimaliaHymenopteraFormicidae

d2864f81-9abf-53ba-bf69-f9166f99ef29

http://zoobank.org/DB5554BA-36B6-4105-988E-DF1D2F284E93

Figures 1 , 2

#### Holotype major worker.

Dominican Republic, “La Toca” mine (ANTWEB1038178) [DZUP].

#### Holotype conditions.

After treatment, the amber piece is now a 15 × 10 × 5 mm, roughly pyramidal structure, glued in a perspex card, and pinned. The specimen presents discrete to moderate distortions in the antennae, mesosoma (especially in the propodeum), legs, waist and gaster. Additionally, head vertexal margin and gaster present abundant compression wrinkles. The inclusion also presents several bubbles, and a smalls internal fractures on the matrix close to the right lateral margin of head which hamper prefect visualization.

#### Diagnosis.

Among the extant *Pheidole* species, †*P.anticua* shares some features with members of the *flavens* group, which is characterized by small size, short antennal scape, thick antennal club, compact body, and vestigial or absent mesonotal convexity. Some of the extant and morphologically similar species are *Pheidolearhuaca* Forel, *Pheidolenitidicollis* Emery, *Pheidoleflavens* Roger, *Pheidolejamaicensis* Wheeler, W.M., *Pheidoleschmalzi* Emery, and *Pheidoletambopatae* Wilson. However, all these species, except for *P.jamaicensis*, present shorter scapes when compared to †*P.anticua*. Additionally, unlike *P.flavens*, †*P.anticua* has a projecting and slightly angulate humerus (like *P. arhuaca, P. nitidicollis, P.jamaicensis, P. schmalzi*, and *P.tambopatae*). Compared with the other five species, †*P.anticua* has different mesosomal sculpture, with a smooth and shiny pronotum but sculptured mesonotum. †*P.anticua* cannot be assigned as the major worker of †*P.primigenia* and †*P.tethepa* due to the absence of humeral spines and the comparatively small body size (considering the average size proportion between *Pheidole* minor and major workers).

#### Measurements

(holotype): HL 0.75, HW 0.71, SL 0.5, EL 0.11, ML 0.63, PSL 0.12, PTW 0.06, PPW 0.15, CI 95, SI 70.

#### Description.

Lateral margins of head, in full face view, slightly convex; with abundant hairs extending laterally. Dorsum of mandible with basal area costate and the remaining surface smooth and shiny. Hypostomal margin straight; with median process vestigial and broad, submedian processes conspicuous, narrow and straight, distant from outer processes. Clypeus, in frontal view, with anterior notch; surface uniformly smooth and shiny. Scape length not surpassing the mid-height between the eyes and the fronto-vertexal lobes; with decumbent to erect hairs. Malar area, in full-face view, with some curved costae near antennal fossae, gradually becoming longitudinal near lateral margins of head. Frons, in full-face view, uniformly costate longitudinally. Antennal scrobe, in full-face view, shallow, internally costate longitudinally, not delimited posteriorly by a curved costulae. Vertexal margin deep, with narrow and strongly convex lobe; surface smooth and shiny.

**Figure 1.**
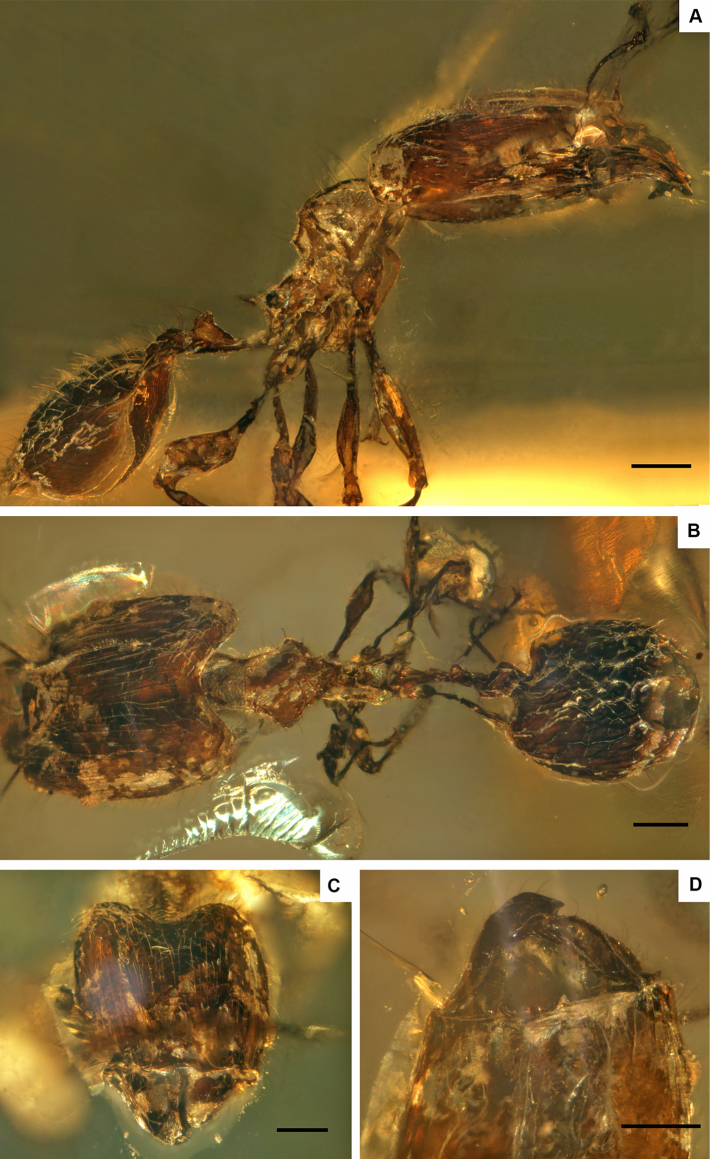
†*Pheidoleanticua* sp. nov. **A** Lateral view **B** dorsal view **C** full face view and **D** hypostomal margin. Scale bars: 0.2 mm.

**Figure 2.**
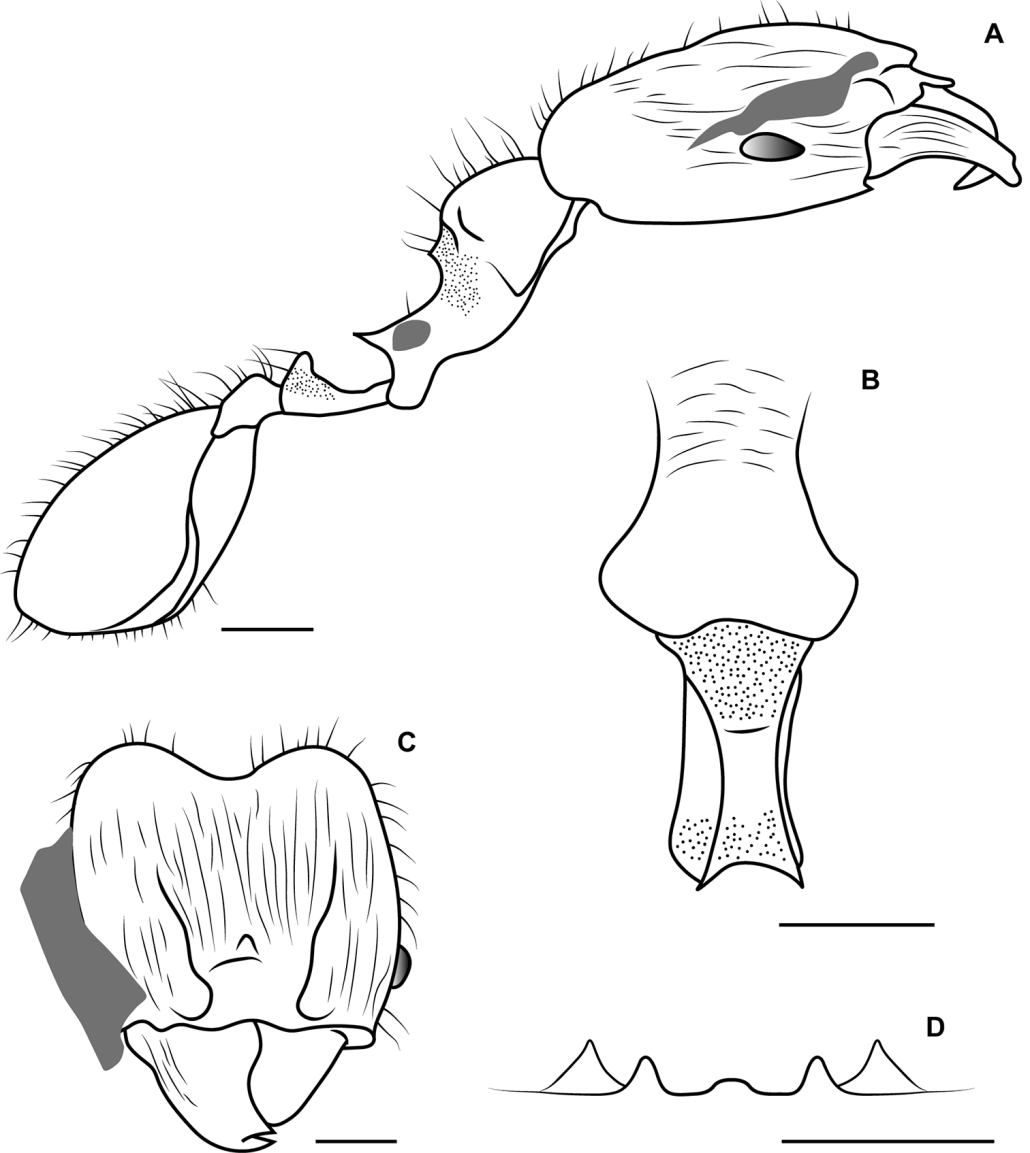
Illustrations of †*Pheidoleanticua* sp. nov. **A** Lateral view **B** full face view **C** dorsal view and **D** hypostomal margin. Scale bars: 0.2 mm.

Humerus, in dorsal-oblique view, projected and slightly angulate; with flexuous hair as long as the adjacent ones. Pronotal profile flat; surface completely smooth and shiny; with abundant long, flexuous and dark hairs. Mesonotum, in lateral view, projected and angulate, abruptly inclined posteriorly; with surface areolate. Katepisternum surface areolate. Propodeum, in lateral view, with long, narrow inclined projections; surface entirely areolate.

Petiolar peduncle, in lateral view, with dorsal margin gradually ascending posteriorly, so that the anterior margin of the node is inconspicuous. Petiolar node, in frontal view, with dorsal margin bilobed; with abundant, long and flexuous hairs, two of which are comparatively longer than the adjacent ones. Postpetiole, in dorsal view, with lateral margins rounded, surface smooth and shiny; with abundant, long and flexuous hairs. First gastral tergum uniformly smooth and shiny; dorsally with flexuous erect to decumbent hairs, less than 1.5× the eye length.

#### Etymology.

From Latin *anticua* meaning old.

## Discussion

†*Pheidoleanticua* is the first fossil species of the genus for which the major worker is described. In Wilson’s (2003) monograph 22 undescribed *Pheidole* fossil specimens are cited, including majors and minors, most of which are deposited in private collections. In the same work is an image of a major worker from the Dominican amber owned by Elizabeth J. Romans and photographed by Frank M. Carpenter (his fig. 6 on p. 11). However, it is impossible to confirm if this specimen is the same as †*P.anticua*, due to low image resolution and not direct comparison.

Castes and subcastes pose a greater challenge to palaeomyrmecology than they do to the alpha-taxonomy of modern taxa. While discussing the taxonomy of extant groups one should refrain from describing a species based on a particular caste. The same procedure is advised for the description of fossil taxa, with specimens sometimes only tentatively associated to a given species (e.g., Baroni Urbani and de Andrade 2003). However, this is not always possible due to the rarity of the material and the difficulty of finding conspecific specimens in the same deposit (e.g., *Pachycondylaoligocenica*Dlussky et al. 2015, described from only a male specimen). *Pheidole* species yet to be discovered in the Dominican amber and from other relatively young New World deposits will most likely suffer from limitations in caste association. We encourage descriptions of majors and minors of *Pheidole*, but we do not recommend descriptions based on males and queens, which will potentially be conspecifics of species already described. Informal descriptions (e.g., “*Pheidole* sp.” of Baroni Urbani 1995, p.12) are highly recommended, as these will add to the knowledge of fossil fauna diversity without artificially inflating the genus taxonomically.

The fossil species †*P.primigenia* and †*P.tethepa* are unique among New World *Pheidole* for having pronotal humeral spines (Wilson 1985; Baroni Urbani 1995), a trait never found in the extant members of the genus in this region. While Wilson (1985) suggested convergent evolution of pronotal spinescence in the Neotropics (and questioned the placement of †*P.tethepa* within *Pheidole*), Baroni Urbani (1995) concluded a relationship between †*P.primigenia* and †*P.tethepa* to Old World spinescent *Pheidole* lineages was more likely. Recent molecular phylogenies have shown that four extant Old-World lineages (*aristotelis*, *quadricuspis*, *quadrispinosa*, and *bifurcata* clades) have independently evolved pronotal spinescence (Sarnat et al. 2017). This suggests that this trait, although uncommon in the genus as a whole, has arisen repeatedly a sufficient number of times to justify Wilson’s hypothesis of convergent evolution of spinescence in the Neotropics.

Among the extinct species of *Pheidole*, the most dubious fossil is †*P.cordata*. Its first record in the literature is Schweigger (1819). In this work, the author listed fossils from Baltic amber and described informally and illustrated an ant with a remarkably large head, showing triangular projections on the propodeum. These projections can be interpreted as propodeal spines or teeth. However, Schweigger did not name this specimen, and some years later, Holl (1829: 140) named it as †*Formicacordata*, using the same characters as Schweigger.

Mayr (1868) transferred it to *Pheidole* (Mayr 1868), even though he believed that Schweigger’s sketch was not clear and Holl’s description was somewhat crude. We conclude that Holl’s decision to describe this species and Mayr’s placement in *Pheidole* may have been hasty. The specimen studied by Schweigger is presumably lost, which precludes its proper placement using current genus concepts in Formicidae (Mayr 1868; Antweb 2019). Dlussky (2008) suggested treating †*Formicacordata* as Formicidae*incertae sedis*, and we concur that there is no strong reason to assume it belongs to *Pheidole*, though it is certainly a myrmicine ant. Thus, we consider †*P.cordata* as *incertae sedis* in Myrmicinae.

Among the fossil ant genera known from Baltic amber, at least two can be associated with †*P.cordata*: †*Stiphromyrmex* Wheeler and *Aphaenogaster* Mayr. Both genera are morphologically very close to *Pheidole* and are characterized by an enlarged head, 12-segmented antenna with a club of three segments, presence of propodeal spines, and a two-segmented waist (Mackay and Mackay 2002; Radchenko and Dlussky 2017).

*Pheidole* was inferred to have originated in the Neotropics at 58 mya with a single colonization in the Old World around 20 mya (Moreau 2008; Economo et al. 2015). Therefore, the presence of a *Pheidole* species in Baltic amber would imply an unexpectedly early dispersal of the genus to the Old World, in the Eocene or earlier. It is also important to highlight that, except for †*P.cordata*, no *Pheidole* species are currently recognized for the Baltic amber, which corroborates the current hypothesis of a New World diversification of the genus with a single event of colonization in the Old Word. In this scenario, we consider †*P.cordata* cannot be safely assigned to any known genus, mostly due to the poor knowledge about its morphology. All definitive fossil records for *Pheidole* are restricted to the New World (Table 1).

**Table 1.** Summary of the *Pheidole* species known from the fossil record.

**Species**	**Deposit**	**Caste**	**Period**
†*Pheidolecordata* (Holl, 1829), *incertae sedis* in Myrmicinae	Baltic amber (34–48 m.y.) (Seyfullah et al. 2018)	Minor worker	Eocene
†*Pheidoletertiaria* Carpenter, 1930	Florissant, Colorado (34.07 ± 10 m.y.) (Evanoff et al. 2001)	Queen	Oligocene
†*Pheidoletethepa* Wilson, 1985	Dominican amber (16–19 m.y.) (Seyfullah et al. 2018)	Minor worker	Miocene
†*Pheidoleprimigenia* Baroni Urbani, 1995	Dominican amber (16–19 m.y.) (Seyfullah et al. 2018)	Minor worker	Miocene
†*Pheidolerasnitsyni* Dubovikoff, 2011	Copal (<1 Ma) (Perkovsky 2016)	Minor worker	Holocene
†*Pheidoleanticua* Casadei Ferreira, Chaul & Feitosa, 2019, sp. n.	Dominican amber (16–19 m.y.) (Seyfullah et al. 2018)	Major worker	Miocene

A question remains regarding the identity of the fossil species *Pheidolerasnitsyni*. Dubovikoff (2011) proposed *Pheidolerasnitsyni* from pieces assumed to be truly Baltic amber, but he latter informed Perkovsky (2016: 117) that they were actually copal. Considering the putative young age of this inclusion (< 1 mya), it is possible that *P.rasnitsyni* is a junior synonym of a modern species. To accurately ensure the identity of this species, direct comparison with extant species would be necessary. However, in addition to the traditional limitations in observing details of morphology in resin inclusions, a second problem is that *P.rasnitsyni* is known only from minor workers. This makes it extremely difficult to determine the relationship between *P.rasnitsyni* and other extant species, since the morphology of minor workers in *Pheidole* is extremely conserved, especially in the Palaearctic species. In this scenario, although we think that *P.rasnitsyni* clearly belongs to *Pheidole*, we encourage a careful analysis of its identity in the future.

## Supplementary Material

XML Treatment for Pheidole anticua
